# Evaluation of multisystemic therapy pilot services in the Systemic Therapy for At Risk Teens (START) trial: study protocol for a randomised controlled trial

**DOI:** 10.1186/1745-6215-14-265

**Published:** 2013-08-20

**Authors:** Peter Fonagy, Stephen Butler, Ian Goodyer, David Cottrell, Stephen Scott, Stephen Pilling, Ivan Eisler, Peter Fuggle, Abdullah Kraam, Sarah Byford, James Wason, Rachel Haley

**Affiliations:** 1 Research Department of Clinical, Educational and Health Psychology, University College London, London, UK; 2 Department of Psychiatry, University of Cambridge, Cambridge, UK; 3 Leeds Institute of Health Sciences, University of Leeds, Leeds, UK; 4 Institute of Psychiatry, King’s College London, London, UK; 5 Islington Child and Adolescent Mental Health Services, London, UK; 6 University of Leeds and South West Yorkshire Partnership NHS Mental Health Trust, Leeds, UK; 7 Centre for the Economics of Mental Health, Institute of Psychiatry, King’s College London, London, UK; 8 MRC Biostatistics Unit, University of Cambridge, Cambridge, UK

**Keywords:** Adolescent, Antisocial behaviour, Conduct disorder, Family, Multisystemic therapy, Randomised controlled trial, United Kingdom, Youth

## Abstract

**Background:**

There is an urgent need for clinically effective and cost-effective methods to manage antisocial and criminal behaviour in adolescents. Youth conduct disorder is increasingly prevalent in the UK and is associated with a range of negative outcomes. Quantitative systematic reviews carried out for the National Institute for Health and Clinical Excellence have identified multisystemic therapy, an intensive, multimodal, home-based, family intervention for youth with serious antisocial behaviour, as one of the most promising interventions for reducing antisocial or offending behaviour and improving individual and family functioning. Previous international trials of multisystemic therapy have yielded mixed outcomes, and it is questionable to what extent positive US findings can be generalised to a wider UK mental health and juvenile justice context. This paper describes the protocol for the Systemic Therapy for At Risk Teens (START) trial, a multicentre UK-wide randomised controlled trial of multisystemic therapy in antisocial adolescents at high risk of out-of-home placement.

**Methods/Design:**

The trial is being conducted at 10 sites across the UK. Seven hundred participants and their families will be recruited and randomised on a 1:1 basis to multisystemic therapy or management as usual. Treatments are offered over a period of 3 to 5 months, with follow-up to 18 months post-randomisation. The primary outcome is out-of-home placement at 18 months. Secondary outcomes include offending rates, total service and criminal justice sector costs, and participant well-being and educational outcomes. Data will be gathered from police computer records, the National Pupil Database, and interview and self-report measures administered to adolescents, parents and teachers. Outcomes will be analysed on an intention-to-treat basis, using a logistic regression with random effects for the primary outcome and Cox regressions and linear mixed-effects models for secondary outcomes depending on whether the outcome is time-to-event or continuous.

**Discussion:**

The START trial is a pragmatic national trial of sufficient size to evaluate multisystemic therapy, to inform policymakers, service commissioners, professionals, service users and their families about its potential in the UK. It will also provide data on the clinical and cost-effectiveness of usual services provided to youth with serious antisocial behaviour problems.

**Trial registration:**

ISRCTN77132214

## Background

This paper describes the protocol for the Systemic Therapy for At Risk Teens (START) trial, a UK evaluation of multisystemic therapy (MST) [1], an intensive family- and home-based intervention for young people with serious antisocial behaviour.

The trial is being conducted at 10 clinical sites across the UK staffed by a team of therapists who have been trained in MST. All sites agreed to participate in a rigorous randomised controlled trial (RCT) as a condition of having a clinical team funded by the UK Department of Health in their locality. Before the launch of the trial, each of the 10 sites providing MST will have been operational in their locality for at least 12 months. This bedding-in period is intended to give each of the sites time to set up local steering groups and raise awareness of their service among referrers (youth offending, social care, child and adolescent mental health, and education services), as well as ensuring that the therapists are adhering to the therapeutic model of MST (see below). Each of the sites will join the trial in phases once all of these conditions are met to a satisfactory level, and the research team will then begin its evaluation. The duration of the evaluation is being funded by the Department of Health, Department for Education and Youth Justice Board.

The need for an accessible, effective technology to manage antisocial adolescents is widely recognised as urgent [2,3]. Youth antisocial behaviour is a common and serious problem with costly consequences for the young people themselves, their families and society in general. In England and Wales, the most recent survey of offending, crime and justice commissioned by the Home Office showed that just over one-fifth of all 10- to 25-year-olds had offended in the past 2 years, and half of these individuals had committed a serious offence, including assault with injury, vehicle theft and burglary [4]. Among younger children, aggressive and disruptive behaviour patterns are associated with peer rejection and school drop-out [5], and longitudinal research has shown that persistent antisocial behaviour in youth significantly increases the risk of criminality, unstable relationships and mental health problems in adulthood [6]. The economic impact of antisocial behaviour is also considerable. Individuals displaying persistent antisocial behaviour at 10 years of age are estimated to cost society 10 times as much as their non-delinquent peers by the time they are 28 years old [7].

The most serious youth antisocial behaviour is committed by a very small group of persistent offenders [8,9]. There is a continuity of disturbance from adolescence to adulthood [10], and those from low socioeconomic status families are most likely to remain antisocial in adulthood. Other negative outcomes include school drop-outs, substance use, unwanted pregnancy, unemployment, convictions, injuries and illnesses, and a two-fold increase in mortality [11-13]. The most antisocial 5% of 7-year-old children are five to ten times more likely to display indices of serious life failure at age 25, for example, drug dependency, criminality, unwanted teenage pregnancy, leaving school with no qualifications, unemployment and so on [14].

In the diagnostic systems developed for classifying mental health disorders of children and adolescents, serious and persistent antisocial behaviour is primarily associated with the diagnosis of conduct disorder (CD). CD is the most common mental health disorder in childhood and adolescence globally, with a lifetime prevalence of approximately 10% [2]. It is the most common reason for referral to child and adolescent mental health services (CAMHS) in Western countries [15,16]. Its prevalence is increasing in the UK [17]. Of juveniles with diagnosable CD, more than three-fifths have severe problems: 29% have pervasive CD with an average of eight symptoms including aggression; a further 29% have on average six symptoms, including theft and other property-oriented offences but not violence; and 3% appear to be primarily aggressive [16]. There is also a pessimistic trajectory from CD in youth to antisocial personality disorder in adulthood [18-21], with young people with the most severe symptoms most likely to progress to antisocial personality disorder [22].

CD and its sequelae place a massive burden on individuals and society, involving not just healthcare services and social care agencies but the family, neighbourhood, schools, police and criminal justice system. Welsh and colleagues [9] estimated the social burden in financial terms of criminal activity of a cohort of 503 typical boys (aged 7 to 17 years), comprising the youngest sample of the Pittsburgh Youth Study of 850 youths in an urban area. Conservatively estimated over 10 years, the cohort caused a substantial burden to society in the form of victimisation costs, ranging from a low of $89 million to a high of $110 million. The group with the most persistent problems, representing 10.2% (*n* = 34) of the offending sample, accounted for half (50.1%) of all self-reported offences, with eight times the average victim costs per offender compared with non-persistent offenders ($793,000 to $861,000 versus $101,000 to $147,000). The cumulative cost to services of individuals diagnosed with CD in their teens over a period of about 18 years was estimated, in 1998 values, to be £70,000 [7]. The cumulative costs attributed to individuals with CD can be about 10 times the costs associated with individuals with other mental health problems [23].

### Multisystemic therapy as an intervention for antisocial behaviour

MST is an intensive family- and home-based intervention for young people with serious antisocial behaviour [1], specifically developed to intervene with adolescents showing serious and persistent antisocial behaviour [24]. It aims to prevent reoffending and out-of-home placements. MST was developed in response to research on the multidetermined nature of serious antisocial behaviour [25], and adopts a social-ecological approach to intervention [26]. The underlying premise of the social-ecological approach embedded in MST is that serious and persistent antisocial behaviour is multicausal; therefore, effective interventions address the multiple sources of offending behaviour that are found not only in the young person (for example, values and attitudes, social skills and biological factors) but also in his or her social ecology (that is, the family, school, peer group and neighbourhood). The key risk factors associated with persistent and serious antisocial behaviour have been identified through decades of longitudinal research and include impulsivity and overactivity on the part of the young person; low levels of parental involvement and harsh, critical parenting; high levels of family conflict and disruption; and the young person’s associations with other deviant peers (for examples, see [27,28]; for a meta-analytic review see [29]). Consequently, in MST, the therapist works primarily with the caregiver to improve his or her parenting skills, enhance family relationships, increase support from social networks, encourage school attendance and achievement for the young person, and reduce the young person’s association with delinquent peers.

MST aims to impact on the entire milieu in which the young person operates, by using multiple interventions, in combinations indicated by the clinical picture. The treatment approach integrates theoretical concepts and techniques from systemic and structural family therapy, parent training, marital therapy, supportive therapy related to interpersonal problems, social skills components, social perspective training, behavioural methods (for example, contingency contracting) and cognitive therapy techniques (for example, self-instructional training). The therapist has an active role as case manager (with typical caseloads of between four and six families), which includes acting as an advocate to outside agencies. MST has been widely adopted in the US and in other countries including Canada, the Netherlands, Norway, Sweden, Denmark, New Zealand and, most recently, the UK.

Recent, high-quality quantitative systematic reviews conducted for two separate National Institute for Health and Clinical Excellence (NICE) guidelines [2,30] have identified MST as potentially the most promising intervention for reducing adolescent antisocial and offending behaviour and improving individual and family pathology (albeit with some heterogeneity in the observed outcomes). There have been 20 randomised trials of MST [31-37], and although the therapy works exceptionally well sometimes [32-36,38], it does not do so consistently [39-42]. In the meta-analytic reviews [2,30], the post-treatment standardised mean difference (SMD) reduction in offending behaviour was −0.47 (95% CI −0.74, -0.21; *P* <0.0001) across seven studies [34-36,43-46]. In eight studies, parent-rated antisocial behaviour was somewhat less consistently reduced with MST compared with treatment as usual (TAU; SMD = −0.25; 95% CI −0.52, 0.02; *P* = 0.07) [33-35,39,43,44,46,47]. On follow-up ranging from 12 to 17 months, based on five studies, the SMD for researcher- or clinician-rated antisocial or offending behaviour was −0.41 (95% CI −0.93, 0.10; *P* = 0.10) [33,36,42,44,48]. Although on continuous outcomes the follow-up yielded an insignificant effect size, on dichotomous outcomes across six studies the reduction of risk was significant (risk ratio = 0.72; 95% CI 0.51, 1.0; *P* = 0.05) [33,42-44,48,49].

The replicability of the findings from the US in other countries has been mixed. In large trials in Canada [42] and Sweden [39], MST failed to reduce antisocial behaviour more than the usual services. Although treatment fidelity may have been lower in these studies than in earlier studies of MST, fidelity scores did not predict outcome, at least in the Swedish study [39].

In the first RCT of MST to be conducted in the US without the direct involvement of the treatment developers, reoffending rates in the MST group remained high even though the intervention significantly reduced reoffending compared with TAU (66.7% versus 86.7%) [49]. Effect sizes associated with efficacy are substantially higher in trials of MST that involved the developers (0.81) than in studies conducted without their close involvement (0.27) [31]. This pattern of results leaves open the possibilities of ‘developer effects’ and that the relative success of MST may be due to the poor quality of the standard services for managing CD in the US. Thus, for MST to be considered valuable, its superiority should be demonstrated outside the US in care systems meeting the following three conditions: the evidence base for TAU (associated with socialised healthcare systems) is stronger than for TAU in earlier clinical trials initiated by the developers of MST (for example, individual psychotherapy); the motivation of the therapists delivering MST to demonstrate favourable outcomes associated with the therapy is lower than that of those who were involved in the development of MST; and sentencing policy within the justice system does not result in a comparison with alternatives, such as custodial sentences.

These three conditions were met with the first UK RCT of MST conducted at the Brandon Centre in London, which evaluated whether MST (*n* = 56) or the comprehensive, targeted services delivered by youth offending teams (YOTs; *n* = 52) was more effective in reducing offending and out-of-home placement in an ethnically diverse, urban sample of young people [44]. MST was provided by a small team of experienced therapists who were not involved with the development of MST and evaluated by similarly independent researchers. The results of this trial showed that, although young people receiving either MST or YOT interventions showed improvement in terms of reduced offending, the MST model reduced the likelihood of non-violent offending during an 18-month follow-up period significantly further. Consistent with the data on offences, the results of youth-reported delinquency and parental reports of aggressive and delinquent behaviours showed significantly greater reductions from pre- to post-treatment levels in the MST group. In this study, MST was observed to have a delayed impact on offending, with between-group differences emerging only at 18-month follow-up. The authors concluded that the superiority of MST over YOT services in reducing offending and antisocial behaviour suggests that MST adds value to current UK statutory evidence-based youth services. It was suggested that the provision of MST would not supplant existing services but would be best used to facilitate appropriate and cost-effective organisation of statutory services for young people and their families.

Although the Brandon Centre trial was a good first step in assessing the appropriateness of MST as a robust intervention for addressing serious antisocial behaviour in the UK, it had several important limitations. First, the sample size was relatively small, and the study was also underpowered to investigate the mechanisms of change believed to be responsible for the success of MST, such as improved parent-youth relationships and decreased affiliations with delinquent peers. Second, the secondary measures used to study mediators and moderators of treatment change were administrated at only two time points - before and after the intervention - and not at the 6-, 12- and 18-month follow-up periods where objective offending data were collected. As a consequence, the nature of, and causes responsible for, the delayed impact of MST on offending could not be ascertained. Finally, the trial examined the effectiveness of MST in youth offenders from a localised population (two boroughs in London) rather than the larger population of antisocial youth that are seen across child and youth services (YOTs, social care, CAMHS) across the UK. The target population and ecological context of the trial is important to consider, as provision of MST in the UK has expanded from one team at the Brandon Centre when the RCT was initiated in 2003 to over 30 teams currently functioning across the country. This expansion is a direct result of a UK government policy decision to help troubled families whose children and young people exhibit truanting and antisocial behaviour.

### The current trial

There are several unresolved issues relating to transporting MST to other health and social care systems and jurisdictions, in addition to the obvious issue that the proposed application (in the context of the current trial) is broader than the targeted samples of previous trials (see below). Additionally, in relation to a UK trial at least, the following issues require systematic monitoring: the clinical competences of therapists to practise MST in the light of curriculum differences for psychologists and social workers in the UK and the US; the stronger evidence base of usual services in the UK than TAUs used in previous clinical trials initiated by the developers of MST; differences in the motivation of MST therapists and the research team who were not involved in its development; contextual issues such as differences in national standards with regard to sentencing policy and practice; the need for appropriate measures and sufficient data for examining mediators (mechanisms accounting for the effects of treatment) and moderators (conditions that qualify the usefulness of treatment) before, during and at the end of treatment; and the need for person-centred statistical analyses to identify typical trajectories of response (for example, for individuals with or without callous-unemotional (CU) traits). It is therefore essential for this major UK trial to include as much detail as possible about the types of YOT and other services made available to young people in the comparator arm, including frequency of contacts with professionals.

START is a national RCT to evaluate MST in the UK context. It has been designed as a pragmatic trial that will inform policymakers, commissioners of services and professionals about the potential of MST in the UK context.

### Aims

The primary aim of START is to establish whether MST is more effective than management as usual (MAU) in reducing out-of-home placement in a sample of adolescents at high risk of being removed from their homes because of antisocial behaviour. Secondly, the trial aims to determine whether the provision of MST leads to improved well-being for young people and their families, including improved emotional and behavioural functioning for the young person, closer family relationships, improved parenting skills, improved educational outcomes and reduced offending behaviour. Thirdly, the trial will establish whether MST is cost-effective compared to MAU and results in reduced costs across mental and physical health, criminal justice and educational systems. Finally, in terms of secondary outcomes, the trial will examine the mediators and moderators of treatment change, and determine whether specific groups of young people presenting with antisocial behaviour benefit to a greater or lesser extent from the intervention. Consistent with extant mediational studies of MST trials with a range of juvenile offenders [48,50], we will test whether the MST theory of change is supported in a broader group of antisocial adolescents in the UK; namely, that MST treatment processes will result in improvement on key family and peer risk factors associated with antisocial behaviour, and that improvements in these risk factors will result in decreased adolescent antisocial behaviour.

In addition, we will improve on previous MST trials, which have rarely examined empirically derived moderators of antisocial behaviour, by studying whether treatment outcome is influenced by the onset of participants’ antisocial behaviour, as well as their level of attention deficit hyperactivity disorder (ADHD) symptomatology and CU traits measured at the beginning of the intervention. Early-onset antisocial behaviour, comorbid diagnoses of ADHD and CD, and high levels of CU traits have been associated with greater severity of antisocial behaviour and poorer youth justice outcomes. For example, early-onset antisocial behaviour has been shown to be the best predictor of early arrest (before the age of 13) and, in turn, young people with an early arrest are the most likely to be chronic offenders by the age of 18 [51,52]. Clinical studies of children with ADHD show that they are at high risk for delinquency [53], and children with comorbid diagnoses of ADHD display more early-onset and severe forms of antisocial behaviour [54]. Research over the past 15 years has demonstrated that antisocial adolescents with high CU traits are more likely to develop severe and violent delinquency over time [55,56], and show more severe and aggressive behaviour [55,57-59]. We propose that young people in the trial showing early-onset antisocial behaviour and high levels of ADHD symptoms or CU traits may benefit less from MST than young people who do not show these characteristics or vulnerabilities.

## Methods/Design

### Trial design

The START trial is a multicentre pragmatic clinical RCT comparing MST with carefully documented MAU for adolescents meeting criteria for being at high risk of requiring out-of-home placement, specifically when this risk is associated with antisocial behaviour, including conviction as a young offender. Whereas previous US trials of MST for youth antisocial behaviour have exclusively recruited offenders from the juvenile justice system, and European trials in Norway [45,60] and Sweden [39] have studied young people served by the child welfare system who have not committed crimes, the current trial will be unique in recruiting a broader range of antisocial young people seen by the range of services and institutions designed to manage antisocial behaviour in the UK. These include YOTs, CAMHS, social services and social care, and the educational system. Consequently, although the common referral criterion is antisocial behaviour, young people will be recruited from various settings where their antisocial behaviour is manifest in different ways (see Inclusion criteria below).

### Ethics

The study protocol was approved by the London-South East Research Ethics Committee (reference number 09/H1102/55). Research and development approval has also been sought and given for each trial site by the relevant NHS Trust in each geographical area.

### Study setting

The study involves each of the 10 MST pilot sites in the UK. Recruitment at each site will take place for 18 months, commencing about 12 months after the inauguration of the programme at each site (sites have begun the programme at different time points), to ensure the embedding of MST at each site before randomisation begins, as described above. At each site, a team of four MST therapists is guided by a supervisor - a doctoral-level (or master’s-level with significant clinical and supervisory experience) therapist with sound knowledge of the theories underpinning MST and their application, and experience in providing clinical supervision. The MST supervisor conducts weekly group supervision, and one-to-one supervision if needed, of the MST therapists at a site to ensure they are adhering to MST principles in their practice. The supervisor is also involved in the selection of young people referred to the service as suitable for MST (see Recruitment and baseline procedures below).

### Participants

Overall, 700 participants are being recruited, with approximately half of the consecutive qualifying cases being randomised to MST alone, and the other half to MAU. The unit of randomisation is the individual participant.

### Sample size and power calculation

**Figure 1:**
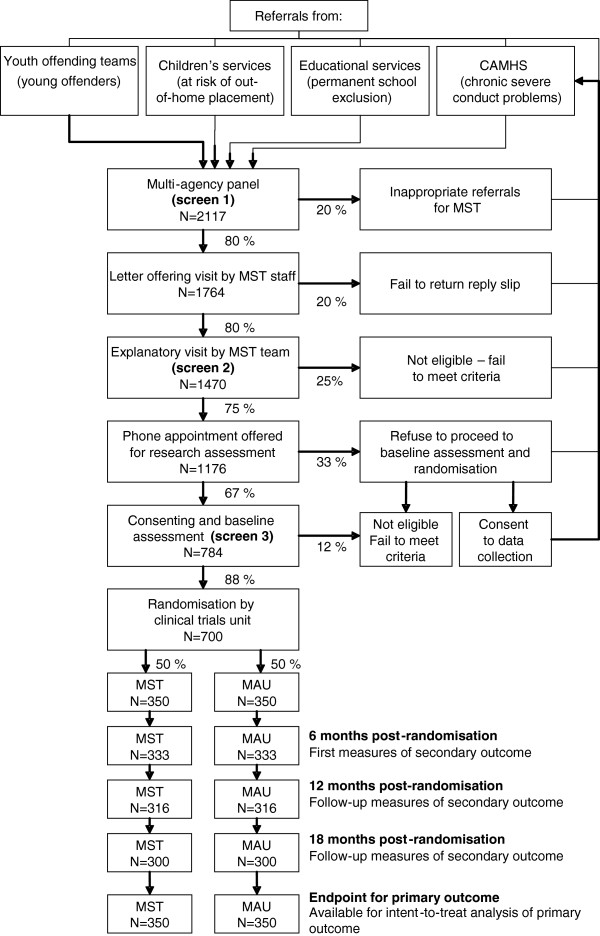
**CONSORT flow diagram of progress through the phases of recruitment and treatment in the START trial of multisystemic therapy.** CAMHS, child and adolescent mental health services; MAU, management as usual; MST, multisystemic therapy.

It is estimated that each site will have at least 140 families per year referred for treatment. On the basis of past experience, data from a meta-analysis of previous trials of MST (including major effectiveness trials from the US) that was carried out in the course of preparation of the NICE guidelines [2], and assumptions outlined below, the investigators assume that about 30% of referred families will meet the criteria and agree to randomisation; this implies that each site will be able to recruit and treat 70 to 80 families during a recruitment window of 18 months. Therefore, investigators expect to be able to recruit and assess 700 participants (350 in each arm). Figure 1 shows the expected flow of participants from recruitment through to the end of the study.

Assuming that 30% of the MAU arm will have out-of-home placements, this sample size will give 86% power to detect a 10% difference in out-of-home placements (a reduction from 30% to 20%). To take account of within-therapist correlation of outcomes in the MST arm, an intraclass correlation of 0.02 and a total of 30 therapists has been assumed, giving design effects of 1.22 in the MST arm and 1.0 in the MAU arm, and thus reducing the power to 83%.

### Eligibility criteria

#### Inclusion criteria

The study design aims to increase generalisability of the findings beyond the group of adolescents under study by using the minimum number of entry criteria. The task of establishing an adolescent target population based on the status of ‘being on the edge of care’ is challenging as, in most contexts, this term covers a heterogeneous group. At the same time, it was recognised that the four referral routes used in the study (YOTs, social care, CAMHS or education services) often identify young people and families with very similar needs when they have reached a particular crisis point. The nature of the crisis may be associated with the specific service (for example, YOTs are triggered by convictions, education services by repeated school exclusions) but the underlying causes in terms of family disorganisation, conflict and stressors, combined with antisocial behaviour by the young person, are similar, and the outcomes in terms of risk of out-of-home placement are common to them.

All participants, regardless of referral source, must meet the following general inclusion criteria:

● Young person aged 11 to 17 years;

● Sufficient family involvement for MST to be applied;

● No existing agency involvement that would interfere with MST (for example, the family is already engaged with a therapist);

● Displaying antisocial behaviour manifesting as at least one of the following criteria indicating suitability for MST:

o Persistent (weekly) and enduring (6 months or longer) violent and aggressive interpersonal behaviour;

o A significant risk of harm to self or to others (for example, self-harming, substance misuse, sexual exploitation, absconding);

o At least one conviction and three warnings, reprimands or convictions in the past 18 months;

o Current diagnosis of an externalising disorder and a record of unsuccessful outpatient treatment;

o Permanent school exclusion.

In addition, participants must meet at least three of the following criteria, which are indicative of risk status and serve as generic indicators of risk sufficient to warrant referral to services:

● Excluded from school or at significant risk of exclusion;

● High levels of non-attendance at school;

● A history of offending, or at significant risk of offending;

● Previous episodes on the Child Protection Register;

● Previous episodes of being ‘looked after’, that is, placed outside of the home (whether via incarceration, psychiatric hospitalisation, residential schooling or assignment to residential local authority care);

● Previous referral to a Family Group Conference (usually a meeting between the family members and sometimes also friends or neighbours, the young person and his/her supporter or advocate if requested, and professionals from the health, education or social services to discuss, plan and make decisions regarding a child at risk to prevent the young person from becoming looked after)

● History of siblings being looked after and taken into local authority care.

Guidelines were developed for inclusion depending on the nature of the referral. The specific inclusion criteria for the four agencies and referral paths are briefly summarised in Table 1.

**Table 1:** Inclusion criteria by referral source in the START trial of multisystemic therapy

**Referral source**	**Inclusion criteria specific to the source**^**a**^
Children’s services	• Designated as ‘child in need’ where this is associated with antisocial behaviour on the part of the adolescent
	• Exhibiting extremely challenging behaviour by either persistent (weekly) *and* enduring (6 months or longer) violent and aggressive interpersonal behaviour *and*/*or* a significant risk of harm to self or to others (for example, self-harming, substance misuse, sexual exploitation, absconding)
Youth offending teams	• At least one conviction within the past 12 months, or referral via a supervision order with multisystemic therapy as a specified activity
	• A warning, reprimand and/or conviction on at least three occasions in the past 18 months
Child and adolescent mental health services	• Current diagnosis of conduct disorder, substance misuse, major depression or anxiety
	• History of at least one unsuccessful outpatient intervention
	• *Either* history of school exclusion *or* assessment as ‘child in need’
Educational services	• Currently permanently excluded from school
	• History of having been excluded from at least one other school for aggressive conduct

^a^All participants must also meet the general inclusion criteria described in the text.

#### Exclusion criteria

The exclusion criteria applied were:

● History or current diagnosis of psychosis;

● Generalised learning problems (clinical diagnosis) as indicated by intelligence quotient (IQ) below 65;

● Identified serious risk of injury or harm to a therapist or researcher;

● Presenting issues for which MST has not been empirically validated, in particular, substance abuse in the absence of criminal conduct or sex offending as the sole presenting issue.

### Recruitment and baseline procedures

The recruitment process is fundamental to the success of this large, multisite research trial. In addition to criteria that apply to recruitment for any trial (for example, the clear application of eligibility criteria, a standard procedure for obtaining informed consent), recruitment for this trial must be especially sensitive to the community context and be based on effective partnerships with referral agencies and strong relationships with the young people and their families. Consequently, the trial team has developed strong collaborative relationships with the MST team at each site to achieve the high levels of accrual necessary to ensure sample comparability and reasonable generalisability.

The authors developed a multiple gating procedure for recruitment based partly on the experience of the MST trial conducted at the Brandon Centre [44]. Using this procedure, decisions about eligibility for the trial have been made at three points: by the multi-agency panel; through discussions between the MST supervisor and trial coordinator on the basis of information gathered from the referral form; and through discussions between the MST supervisor and trial coordinator following gathering of initial information by the clinical and research teams after an introductory home visit to explain the rationale for the research trial and answer any questions about the clinical services potentially available to the family. Experience suggests that each of these screens tends to reveal different criteria for ineligibility, and their use in combination minimises the (considerable) effort of recruitment. For example, the multi-agency panel review tends to identify referrals for issues for which MST has not been empirically validated (for example, sex offending as the sole presenting issue), whereas discussions between the MST team and family or referral source most commonly identify a risk of possible injury to a worker or agency involvement precluding inclusion in the trial.

Finally, the research assessor’s (RA) assessment is necessary to confirm the young person’s IQ and psychiatric diagnosis.

#### Screen 1

Multi-agency panels have been put in place at each of the 10 sites, taking referrals from YOTs, CAMHS, social care and education services. These panels identify new cases that meet the eligibility criteria for MST. In addition, the MST team at each site directly takes referrals, as they have become established and connected with other services and referral sources in each of their localities. The decisions made by the multi-agency panels and the MST team constitute the first screen. During the period between establishment of the teams and the start of the trial, each MST team gained experience in screening referrals and educating potential referrers as to what constitutes an appropriate referral to the trial. A standard referral form for each locality is used in liaison with the research team, including specific information pertaining to inclusion and exclusion criteria. This forms the first screen to the trial, focusing on establishing the severity and chronicity of antisocial behaviour.

#### Screen 2

Following acceptance of the referral by the multi-agency panel, a standard initial letter from the clinical team, in the first language of the family, is sent to the parents and separately to the young person if aged 16 or over, informing them about the trial. Recruitment begins with this letter, which is sent to all those identified by a panel and/or MST team to be probably suitable for MST. The letter invites participation, and informs the family and the young person that someone from the MST team (preferably the MST supervisor) will contact them unless they do not wish to participate in the trial. The letter includes material in age-appropriate and culturally appropriate language in the form of a leaflet for both the parents and the young person explaining the trial and what involvement in the trial would mean at that particular site. These information sheets have been constructed and designed with input from young people and their parents or carers who have used MST at the same site to make them as useful and accessible as possible.

After 3 to 7 days the family is visited by a member of the MST team to explain what participation might involve and to arrange an appointment at the family home that will include the MST supervisor or therapist, the RA, the young person and his or her primary carer(s). The discussions at this meeting include the identification of an acceptable and credible MAU path (the best available alternative treatment at that locality; see below) should the young person not be randomised to receive MST.

At this meeting, which constitutes the second stage of the multiple screening procedure, the MST therapist identifies possible exclusion criteria, such as a risk of injury or harm to a worker, agency involvement incompatible with participation in the trial, or severe substance dependency. Consent forms are not signed at this appointment because experience has shown that actual take-up of treatment increases dramatically and early drop-outs are reduced if the family feels they have been given adequate time to consider participation.

#### Screen 3 and consent

Unless the family expresses a decision not to participate at the time of the visit or within 3 to 7 days after the visit, a telephone contact is made by the RA, offering a research assessment. At this stage the consent form is reviewed. Consent forms signed by both the young person and carer(s) include permission to access the police and correctional databases, remaining in effect for 3 years. For families who sign the consent forms, the RA administers pre-randomisation questionnaires and measures are completed during this contact (that is, before group assignment). The final evaluation for eligibility (the third screen) is made at this second visit.

When all the instruments have been completed and eligibility for the trial confirmed, the RA telephones the trial centre to inform the centre of the family’s randomisation details. The randomisation is performed and details communicated to the referrer and family within 24 hours.

#### Randomisation and procedures to minimise bias

After consent has been obtained and the baseline assessment has been carried out, a trial identification is assigned. Eligible consenting participants are randomised to MST or MAU on a 1:1 basis by the Trials Unit at University College London using a secure telephone randomisation service that ensures allocation concealment. A computer-generated adaptive minimisation algorithm that incorporates a random element is used with the following stratification factors: treatment centre, gender, age (11 to 14, 15 to 17) and age at onset of conduct problems (2 to 11, 11+). These strata were selected because previous research has shown that a younger age of onset is associated with poorer prognosis; there is insufficient evidence concerning gender mix, particularly in the older age groups, to ensure that simple randomisation would generate comparable groups. Treatment centre is included in the minimisation stratification to control for differences between centres.

To minimise bias that could arise from knowledge of treatment allocation, the following strategies are employed: RAs are blind to treatment allocation; the RA and therapist do not communicate directly (if at all) with each other; qualitative interviews, conducted with a subsample of the families by doctoral students trained and supervised by experienced qualitative researchers, are audiotaped and a random sample re-rated by independent raters. The qualitative interviewers are separate from the MST service providers.

### Planned interventions

#### Multisystemic therapy

There are a number of factors that contribute to the unique nature of MST: it addresses the multidetermined nature of severe conduct problems; it sees the caregiver as key to effective behaviour change; it uses the adolescent’s home as the primary site of the intervention; it integrates several evidence-based intervention techniques; it uses a single therapist to deliver multiple modalities of intervention within a singular conceptual framework; and there is rigorous monitoring of adherence to the model, including the use of comprehensive quality assurance and on-going quality improvement system for maintaining standards.

MST is a family- and community-based intervention that uses intense contact with a family to understand and address the drivers of a young person’s antisocial behaviour. It targets drivers related to the young person’s individual adjustment, family relationships, school functioning and peer group affiliations. A focus on the caregiver and family is key to the intervention. MST interventions are individualised and highly flexible but are documented in treatment manuals [61]. The package is designed to work with hard-to-reach families in the community and provides a duty cover system 24 hours a day, 7 days a week to ensure that families receive support from the MST team at the times when crises are occurring. Although the package comprises a number of evidence-based interventions, it is designed to be delivered by one highly trained professional, typically a mental health professional with graduate training in one of a variety of disciplines (for example, clinical psychology, social work) with a caseload of four to six families. The MST therapist is a full-time generalist who directly provides most of the mental health interventions and directs access to services, coordinating these to monitor quality control. The therapist is available to the family 24 hours a day, 7 days a week but input is adjusted according to need. Treatment fidelity is ensured by weekly group supervision meetings between therapists and the MST supervisor; these meetings may also include a medical consultant. Treatment generally lasts 3 to 5 months. Sessions are held in the family’s home and in community locations [61]. Especially in the beginning, the therapist may be in the home three times a week and may speak to the young person’s parent or principal carer on days when no visit has been arranged. The MST therapist also spends time at the young person’s school as needed and meets with the young person’s peer group and extended family. A key early part of the process is to engage the family, a significant challenge in some cases.

Despite incorporating a range of modalities, MST is more than just an amalgamation of techniques and approaches, and a focus on the inter-relationship between social, education and healthcare systems is retained. The overarching goals of MST are to give parents the skills and resources needed to address the inevitable difficulties of raising adolescents, and to empower adolescents to cope with familial and extrafamilial problems. Assessment and treatment explore the adolescent’s role in various systems and consider the inter-relationship between these systems. Specific attention is given to strengthening the various systems, and an attempt is made to promote appropriate and responsible behaviour among all family members. MST focuses on current behaviours as opposed to influences from the young person’s past. It seeks to identify, reduce and, if possible, remove behaviours that are of concern to the referring service and the young person’s parent or principal carer. The implementation of MST draws on family members, in contrast to many interventions that define the young person as the ‘identified client’. In addition, collaboration with community agencies is a crucial part of MST. For instance, establishing (or in most cases re-establishing) engagement between the young person, the parent(s) and the school is a critical part of the treatment, and the MST therapist may be in regular contact with teachers. The MST therapist also works in close partnership with the social worker from the YOT or social services, who may have statutory responsibilities for the young person. When considered necessary, the young person is referred to a general practitioner or other medical professional who can assess the young person and prescribe medication.

As mentioned above, the MST teams are centrally funded by the Department of Health and had already been operational for approximately 12 to 18 months prior to their participation in the START trial. These factors benefit the RCT in several respects. First, at the start of the trial the clinical teams had already developed relationships with key stakeholders and referrers in their local communities, and multi-agency panels had been in operation to facilitate recruitment. Second, the clinical teams had already had the opportunity to liaise with potential sources of referrals regarding the appropriateness of referrals, and thus to ensure that referral of inappropriate cases is minimised. Third, as the funding of the MST teams is tied to participation in the research trial, the collaboration between the MST clinical teams and the research team to achieve common goals (for example, recruitment numbers, implementation of randomisation, completion of the research protocol) is maximised. Finally, by the start of the trial, the therapists and supervisors would have been able to develop experience implementing MST; research suggests that mature MST teams are associated with greater adherence of therapists to the MST model. The association between treatment fidelity and outcomes for adolescents has been repeatedly demonstrated in trials of MST [35,39,48,50]. To mitigate the dissipating influence of poor adherence, the MST developers have developed an extensive quality assurance system [62]. In common with all MST sites, the 10 START trial sites were licensed by MST Services Inc. (Charleston, SC, USA), and therefore participated in MST Services’ quality assurance procedures. As well as the weekly supervisions with the MST supervisor, the therapists receive a one-hour weekly telephone consultation with an MST expert, on-site booster training sessions four times per year, and twice-yearly implementation reviews by the MST expert. The supervisor guides clinical work according to the MST Supervisory Manual, and in delivering MST the trial team adheres to the MST Organizational Manual [63].

#### Management as usual

MAU consists of the standard care offered to adolescents and their families who meet eligibility criteria for the trial in each of the sites. This treatment is likely to be diverse and may involve no therapeutic intervention or individual- or family-oriented work. It is likely to be delivered by a wide range of practitioners with different theoretical orientations. The average duration of these interventions is also likely to vary. It is expected that practitioners will be working in line with best practice as specified in relevant Social Care Institute for Excellence and NICE guidance [2,30].

Based on assessments, the young people in the MAU group receive a tailored range of interventions aimed primarily at preventing reoffending. As in MST, typical interventions are extensive and multicomponent, consistent with the complex mental health needs of this population in the UK [64]. Interventions include helping the young person to re-engage in education; help with substance misuse problems and anger management; social problem-solving skills training; and awareness programs relating to vehicle crime, violent offending and knife crime. It is likely that victim awareness and reparation interventions will be included as appropriate. The treatments are delivered by professional social workers, specialist therapists or probation officers.

The key differences between MST and MAU are that MAU interventions are not normally delivered in a family context by a single person. Typically, no overarching model governs the selection of treatments, and there is no set of principles comparable to that of MST to organise the therapies offered. Rather, interventions are likely to be offered on an ‘as needed’ basis by specialist agencies to which the young person is referred. The young people allocated to MAU are likely to receive considerable attention from a range of professionals delivering evidence-based protocols. In a recent RCT comparing MST with usual services provided by YOT, clients typically received around 21 professional appointments over the period that MST was administered [44,65]. It is unlikely that practitioners in MAU receive the extent or quality of supervision available for MST therapists. However, it is by no means clear that, even during the trial period, MAU interventions are likely to be less intensive or less costly than MST, although they are likely to be delivered in a less focused and less well-specified manner, with greater variability in service provision. In the Brandon Centre trial [44], young people in the control arm attended significantly more appointments than those in the MST arm. This was in line with the National Standards set out by the UK Youth Justice Board for the key contact and enforcement community [66].

For this reason, MAU interventions in the current trial are carefully monitored using a service use schedule designed specifically for the trial, which records contact with all services (health, social, YOT, education, voluntary sector, and so on), including the number of contacts (and possibly the average duration of contacts). This will give a realistic sense of the level of intensity of MAU in whatever form, and, used in conjunction with the MST arm, will give an indication of shifts in intensity - that is, whether the addition of MST reduces the need for other support. As START is a pragmatic trial involving a number of collaborating services even within each individual site, it was not possible to specify in advance what MAU would constitute.

### Assessments and outcome measures

To maximise the clinical validity of the outcome evaluations, assessments are being made across multiple domains using multiple methods and sources.

#### Primary outcome

The primary outcome is the proportion of cases assigned to long-term (3 months or longer) out-of-home placements in specialist residential provision, including placement into local authority care, incarceration, long-term hospitalisation and residential schooling, at 18 months following randomisation. The investigators expect this trial to give information on how many children assigned to MAU and MST require specialist residential provision either immediately or during the follow-up period.

#### Secondary outcomes

The number of secondary outcomes is limited to reduce the measurement burden of the study, which was found to be a disincentive to continued participation in the Brandon Centre study [44]. The domains that the investigators consider key to the intervention are youth offending outcomes, adolescent well-being outcomes and family functioning outcomes. In addition, the economic data collected alongside these outcomes are considered highly important. The study is also designed to collect data on variables that target key mechanisms of change identified in previous MST studies - parent-adolescent relationships and young people’s associations with deviant peers - and to evaluate parenting skills in detail, given that MST aims to improve young people’s lives by targeting caregivers as being primarily responsible for facilitating change. Adolescent symptoms have been shown to decrease in association with increased supportiveness and decreased conflict between parents [67,68] and with an increased caregiver follow-through on discipline practices [69]. Further, adherence to the MST manual by therapists appears to improve family functioning, which in turn decreases deviant peer affiliation, leading in turn to decreased delinquent behaviour [50].

#### Objective measures

Objective secondary outcomes will be collected from reports of offending behaviour based on police computer records, including details of custodial sentences. These measures will be taken at 6-monthly intervals, for the 6 months before randomisation, the 6 months covering the intervention period, and 6-monthly until the 18-month follow-up point. The number of records of offending behaviour (count data) will be obtained and 6-month periods free of any offending behaviour will also be recorded (binary data). Records are obtained from the Police National Computer as well as from the Young Offender Information System database at each site; these records detail information on offences, court appearances, criminal orders, police custody records and arrest rates.

Records of school attendance and exclusions will be retrieved from both the local schools themselves and the National Pupil Database to assess educational outcomes.

#### Self-report

The RA administers pre-testing questionnaires during the initial contact with the young person and family after they have given consent to participate in the trial, prior to group assignment (as described above). Post-testing by the RA is scheduled for 6 months after entry into the study; it is envisaged that this will be a minimum of 2 weeks after the family completes the intervention. Follow-up assessments will be made at 12 and 18 months post-randomisation.

Self-report measures of well-being and adjustment, antisocial behaviour, parenting skills and family functioning as well as parental mental health and adjustment are collected.

*Well*-*being and adjustment*. A general assessment of well-being is taken using the Strengths and Difficulties Questionnaire [70]. Educational outcomes will include evaluation of participants’ emotional and behavioural functioning in the classroom using the Conners Comprehensive Behaviour Rating Scale-Teacher report form [71]. Depression will be monitored using the Short Mood and Feelings self-report questionnaire [72], completed by the youths.

*Antisocial behaviour*. The prevalence and incidence of delinquent behaviour such as vandalism, theft and burglary will be monitored using the Self-Report Delinquency measure [73]. Noncompliance and increasingly serious forms of antisocial behaviour, together with young people’s perceptions of law-abiding behaviour and institutions, will be measured using the Antisocial Beliefs and Attitudes Scale [74]. Peer delinquency will be assessed using the Self-Report Delinquency measure [73]. The investigators predict that MST will achieve decreases in associations with other antisocial peers, increases in positive peer relations and greater commitment to prosocial activities (for example, education). This prediction is consistent with the model and hypothesised mediating mechanisms [50] and is relevant to social policy initiatives and concerns.

*Parenting skills and family functioning*. The quality of the parent-adolescent relationship, family functioning and parenting practices will be evaluated using the Family Adaptability and Cohesion Scales [75] and the Alabama Parenting Questionnaire-Short Form [76]. Parental disruption will be assessed using the short form of the Conflict Tactics Scale [77], and the level of expressed emotion in the home (as conceptualised in the Camberwell Family Interview) will be assessed using the Level of Expressed Emotion questionnaire [78,79].

*Parental mental health and adjustment*. A brief assessment of parental mental health will be obtained using the commonly used screening instrument, the General Health Questionnaire-28 [80].

#### Interviews

*Demographics interview*. A bespoke interview (Demographic Interview for Parents) to cover general family information, including parental forensic history, schooling and economic information, has been constructed and will be administered to all parents. Child psychometrics will be obtained from the Wechsler Abbreviated Scale of Intelligence [81].

*Psychiatric screening*. Psychiatric disorders will be identified and a psychosis screen provided by the Development and Well-Being Assessment [82]. This computerised structured interview measure is administered to both the parents and young person.

*Experience of MST*. Finally, young people and their carer(s) will be interviewed to elicit their views regarding their experience of MST and factors that they perceive had facilitated or inhibited therapeutic change, based on promising qualitative research carried out during the Brandon Centre trial.

#### Health economic evaluation

Health economic analysis will be conducted by the Centre for the Economics of Mental Health at the Institute of Psychiatry, London, to explore the relative costs and cost-effectiveness of MST and MAU. The evaluation will take a broad perspective, including all health, social services, education and voluntary sector services, plus costs to the criminal justice sector, costs resulting from crimes committed, and out-of-pocket expenses to the young people and their families.

Data on MST contacts will be collected directly from the pilot schemes to avoid participants revealing their group allocation to the RAs. Data on the use of all other services will be collected in interviews using the Child and Adolescent Service Use Schedule previously used with young people with complex mental health and social care needs [83-86]. The Child and Adolescent Service Use Schedule will be adapted to the current study population by review of the literature and pilot testing, to ensure comprehensive coverage and face validity. The cost of the trial interventions will be calculated through a detailed micro-costing (or bottom-up) approach using standard costing methodology [87,88], which will involve estimation of the indirect time spent on individual cases, including preparation, meetings, telephone calls and supervision, as well as detailed recording of direct face-to-face contact.

### Moderators of outcomes

#### Therapist adherence to the multisystemic therapy model of intervention

The MST Therapist Adherence Measure-Revised (TAM-R) measures the team’s adherence to the nine MST treatment principles [50]. The TAM-R is a 28-item measure completed by parents or primary caretakers, often administered by the MST team. In the START trial it will be administered independently from the MST team by an RA not associated with that site or the families working with it. Data will be collected from the second week of MST treatment and every 4 weeks thereafter. Research has demonstrated associations between therapist adherence to the MST principles as measured by the TAM-R and youth outcomes [35,39,48,50,89-91].

#### Psychopathic traits and attention deficit hyperactivity disorder

The trial will also evaluate potential moderators that have not been widely explored in previous studies because of sample size constraints, and will consider in particular the role of psychiatric comorbidities including psychopathic traits and ADHD. Young people with high levels of psychopathic traits show a greater likelihood of recidivism [92], and one study found that young children high for CU traits showed poorer outcomes in a parent training programme for children [93]. It is well established that the combination of ADHD and antisocial behaviour is associated with greater amounts of physical aggression, a greater range of persistence of antisocial activity [94] and the persistence of antisocial outcomes in adulthood [95]. Psychopathic traits will be assessed using the Inventory of Callous-Emotional Traits [96], and the Diagnostic and Statistical Manual of Mental Disorders 4th edition ADHD subscales from the Conners Comprehensive Behaviour Rating Scale [71] will be completed by parents and teachers to assess hyperactivity and impulsivity across home and school settings. ADHD diagnoses will also be arrived at categorically using the Development and Well-Being Assessment (see above).

### Programme components of multisystemic therapy and management as usual

A bespoke fidelity measure has been developed to enable accurate characterisation of the programme components of MST and MAU interventions, the Children and Young People – Resources, Evaluation and Systems Schedule (CYPRESS) (S. Pilling, S. Butler, C. Gaffney, P. Fonagy, personal communication). This approach is based on the initiative of the MST developers [62], who have undertaken a large-scale transportability study with 2,000 families, 429 therapists, 122 supervisors and 20 consultants. Organisational structure and climate as rated by the therapists, supervisors and consultants appears to predict the outcome of MST [97]. CYPRESS was designed to characterise care pathways for antisocial youths in a UK context. It will be administered as an interview to service managers and therapists and will elicit care-pathway-relevant information, for example, in relation to service characteristics, team operations, and the range of interventions available to young people and their families. CYPRESS will allow the identification of key programme elements associated with outcome. More specifically, the use of a common measure will allow comparison between MST and MAU along important dimensions of care and potentially provide information on common aspects of service function associated with outcome. Such an approach has been used to characterise other complex interventions in mental health [98]. It will be of particular value in assessing the key beneficial organisational components of such a complex intervention as MST when it is deployed in a healthcare system radically different from that in which it was developed [99].

### Qualitative interviews

To obtain additional information about the experience of MST and being in an RCT of this treatment, the semi-structured interview developed by Tighe and colleagues in the Brandon Centre trial will be used [100]. This 45-minute interview elicits parents’ and young people’s experience of MST or control treatments, and their views of the costs and the benefits. Semi-structured interviews will be conducted to explore how young people and their parents or caregivers experienced MST, whether they think that the young person’s functioning or carers’ lives have changed following MST (in areas targeted by the intervention), and whether family relationships have changed. If the young person or parent reports changes, the interview will also explore the timing of those changes and how MST affected the changes.

Interviews will be audio recorded and transcribed verbatim. Transcripts will be analysed using framework analysis, a structured method for analysing themes in data, developed by Ritchie and Spencer [101], which facilitates the systematic analysis of large amounts of qualitative data [100]. The researcher develops a structured thematic coding framework for the data, which is then used to systematically record the occurrence of each of the categories in the entire data set. Charts are then developed to show the pattern of occurrences of each theme, and these are used to map and interpret the data set as a whole.

The intention is for the interview to be administered to a random 5% of families across all sites by a person independent of the intervention but not blind to treatment allocation.

### Follow-up assessment

Follow-up assessments will be conducted at 6, 12 and 18 months post-randomisation. Table 2 shows a detailed outline of the planned measures at each follow-up point throughout the trial.

**Table 2:** Assessments and schedule of administration in the START trial of multisystemic therapy

**Assessment**	**Timeline (months)**			
	**Baseline**	**6**	**12**	**18**
**Eligibility and consent**				
Eligibility assessed by MST panel	x			
Consent taken at introductory visit	x			
Randomisation information provided	x			
**Questionnaires-Parent**				
Child and Adolescent Service Use Schedule (excluding last two questions for young person)	x	x	x	x
Demographic Interview for Parents	x			
General Health Questionnaire-28	x	x	x	x
Conners Comprehensive Behaviour Rating Scale-Parent form	x	x	x	x
Inventory of Callous-Unemotional Traits	x	x	x	x
Self-Report Delinquency	x	x	x	x
Development and Well-Being Assessment	x		x	
Conflict Tactics Scale-Short Form	x	x	x	x
Alabama Parenting Questionnaire-Short Form	x	x	x	x
Family Adaptability Service Use Schedule-Caregiver Questionnaire	x	x	x	x
Loeber Caregiver Questionnaire	x	x	x	x
**Questionnaires-Young person**				
Child and Adolescent Service Use Schedule (last two questions)	x	x	x	x
Short Mood and Feelings Questionnaire	x	x	x	x
Inventory of Callous-Unemotional Traits	x	x	x	x
Self-Report Delinquency	x	x	x	x
Level of Expressed Emotion	x	x	x	x
Wechsler Abbreviated Scale of Intelligence	x			
Antisocial Beliefs and Attitudes Scale	x	x	x	x
Strengths and Difficulties Questionnaire	x	x	x	x
Development and Well-Being Assessment	x		x	
Alabama Parenting Questionnaire-Short Form	x	x	x	x
Youth Materialism Scale	x	x	x	x
EQ-5D^a^	x	x	x	x
**Education data**				
Conners Comprehensive Behaviour Rating Scale-Teacher form	x	x	x	x
Attendance/Exclusion rates	x	x	x	x
**Youth offending data**				
Offending history	x	x	x	x
**Interviews**				
Optional Qualitative interview - parent or carer				x
Optional Qualitative interview - young person				x

^a^EQ-5D is a measure of health outcomes (used in the health economic evaluation).

### Statistical analysis plan

All analysis will be according to the intention-to-treat principle. The characteristics of the treatment groups will be described at baseline. Preliminary analysis will investigate the pattern of missing follow-up data.

#### Primary outcome

The primary analysis will be a logistic regression with random effects to account for clustering by therapist. The analysis will include centre, number of past convictions, gender, age at onset of criminal behaviour and other risk indicators as fixed effects. The logistic regression will be fitted using generalised estimating equations. A Wald test of the effect of intervention will be used as the primary analysis. As a secondary analysis, tests of interaction will be used to explore whether the intervention differs according to gender, age, presence of a criminal record or referral path. Clustering by therapist will be accounted for by computing robust standard errors [102].

#### Secondary outcomes

The antisocial behaviour outcome (time to offence) will be analysed using a Cox regression. All other secondary outcomes will be modelled using linear mixed-effects models, with separate treatment effects for the 6-, 12- and 18-month outcomes and an unstructured covariance matrix. The effect of intervention on the 18-month outcome will be tested using a Wald test. Tests of interaction will be performed for all secondary outcomes in which a nominally significant treatment effect is found.

It is anticipated that the primary outcome will have very little missing data as these data are obtained independently of the participants. For the secondary outcomes, linear mixed models and Cox regression yield valid inferences when data are missing at random (that is, the probability of a particular data point being missing depends only on observed data). It is possible that data may be missing not at random, so sensitivity analyses will be conducted to explore the impact of missing data.

There is just one primary outcome, so the primary analysis will be conducted at a nominal two-sided significance level of 0.05. There are several secondary outcomes, so caveats will be used when interpreting effects of treatment on secondary outcomes. No formal correction for multiple testing will be used.

There is no plan to analyse outcomes by site and it is hoped that TAM-R scores will pick up the variance associated with individual therapists.

## Discussion

Although a number of high-quality efficacy studies have demonstrated the success of MST in reducing antisocial behaviour and out-of-home placements in high-risk youth, the replicability of the original findings from the US when MST has been transported internationally has been mixed, especially when field trials have not involved the developers of the programme. Although the evidence from the US suggests that MST is a very promising treatment, the question of whether it will be similarly effective in the UK has not been fully tested. Substantial attention has been paid in past research to treatment transportability and fidelity, but variability in the outcomes of different studies may be due to the comparison conditions not being adequately comparable across studies. The provision for antisocial adolescents in the UK is difficult to compare with that in the US, and expectations of favourable outcomes relative to MAU are not based on comparable investigations. The sole UK study, unlike counterparts in the US, found statistically significant reductions in non-violent criminality only after 18 months following randomisation [44]; however, this was an underpowered investigation with limited measures of critical outcomes such as self-reported criminal behaviour and observational or self-report studies of mental health and well-being. To assess the efficacy of MST in the UK context, the START trial will compare MST with the multi-agency interventions that are currently provided for these adolescents through the UK National Health Service by specialist YOTs and by social services and education services following recommendations by the Social Care Institute for Excellence and NICE. To ensure that the study achieves the fairest and least biased assessment of the potential benefits of MST, the investigators have designed the study with the support of MST Services but will carry out the RCT independently from the developers; however, the developers have access to the trial sites to ensure that treatments are delivered at the highest levels of fidelity [97].

The research is being conducted across 10 pilot sites, each overseen by a team of therapists who have received specialist training in MST to ensure high-quality delivery of the intervention. As out-of-home placement (whether incarceration, hospitalisation, residential schooling or assignment to residential local authority care) in the majority of cases represents an unhelpful outcome for antisocial adolescents, the investigators have chosen to use preservation of the family as the main measure of benefit. As noted above, the rate of out-of-home placement is an important primary outcome, but it is not in every instance an indication of the failure of the system to provide adequate support to the young person and family. Consequently, the authors intend to interpret the information on family preservation in the context of combined information collected from measures of family functioning and home observations to categorise treatment successes and failures regarding out-of-home placement according to pre-assigned criteria. In doing so, it will be possible to capture instances where out-of-home placement is an appropriate outcome and when it is not; for example, instances when there is no out-of-home placement but home observation data and self-report measures suggest that the young person’s situation remains markedly suboptimal.

MST aims to reduce the level of offending in the target population, and so offending rates will be used as a key measure to determine whether the intervention is effective. Other possible benefits of MST will also be examined, such as the impact on the young person’s educational progress, his or her mental health and well-being and that of the parents. To address the mediators of improvement, the investigators have taken considerable effort to monitor the effect of MST on parenting and family function. It is anticipated that improvement observed in association with this intervention will be commensurate with the impact that MST has on variables within the family, and for other clinically critical outcomes (for example, antisocial peer group affiliation), to be in line with changes in the purported mediating processes.

To assess what the benefits of MST and MAU might be, care will be taken to describe the interventions delivered to both groups in the study accurately, and the investigators will attempt to chart the subjective experience of all stakeholders in the project (that is, service users and providers and commissioners of services). The data from the trial will be analysed to determine whether the expected benefit of MST is achieved and whether it represents an economically viable option.

The START trial is a pragmatic national RCT that is of sufficient size to evaluate MST in the UK context to inform policymakers, commissioners of services, professionals, service users and their families about the potential of this treatment in the UK. Given the number and geographical spread of sites in the MAU condition, the trial will also provide unprecedented data on the clinical effectiveness and cost-effectiveness of usual services provided to young people with serious antisocial behaviour problems.

The START trial intends to recruit a large sample of 700 young people and their families, who will be evaluated repeatedly over a period of 18 months. This will allow their trajectories of adjustment to be mapped over an extended period following the intervention. Moreover, the large sample size, combined with the study design and measurement strategy, will provide for a detailed investigation of mechanisms related to treatment response. The investigators are able to build on earlier studies that have found theoretically consonant mediators [50,67-69]. Few moderators beyond treatment fidelity have been addressed in the literature on MST. The Norwegian trial of MST [60] showed that although girls presented a slightly different problem profile from boys, MST appeared to be robust to gender. To date, no studies have examined in depth, with a large sample, which types of young people benefit most or least from MST. For example, there are outstanding issues regarding whether MST is equally effective across the diverse range of ethnicities in the UK, as well as with the emotional comorbidities associated with antisocial behaviour such as anxiety and depression (the latter particularly in females). Ethnic match between therapist and youth has been observed to be associated with short-term treatment effectiveness [103] but not long-term criminal outcomes [104]. One small-scale study contrasted the outcomes of MST in youths presenting with pure externalising problems versus those with mixed internalising and externalising problems [105]. Youths with mixed presentations benefited somewhat less; in addition, the extent of their change in clinician-rated externalising behaviour was associated with maternal depression. In post hoc analyses, with appropriate caveats, this study will permit starting the process of identifying the specific subgroups of youth who uniquely benefit from MST as opposed to MAU. This will have obvious practical benefits to services which have to treat a large, heterogeneous group of youth showing antisocial behaviour.

The investigators will undertake several initiatives that are essential to understanding the impact and cost-effectiveness of MST in the UK. Building on an initial qualitative study of MST in the Brandon Centre trial [44], young people and their carers will be interviewed to elicit their views on receiving this intensive, ecologically valid intervention and, specifically, to learn what factors they identify as having facilitated or inhibited treatment change. The qualitative interviews provide an opportunity to collect outcome data that can then be triangulated with the relevant and comparable quantitative outcome measures. This entails a process of interpretation whereby quantitative findings regarding the primary and secondary outcomes will be complemented with participants’ descriptions of processes that they believe contributed to improvements or the absence of desired change. As an illustrative example, qualitative findings from the Brandon Centre trial showed that parents identified their son’s continued association with deviant peers as an important factor when they continued to engage in criminal behaviour. The investigators will also undertake to carefully define and track what services are given in the MAU condition, and to study the organisational or service factors that may contribute to outcome. Finally, a comprehensive cost-effectiveness evaluation will be undertaken to look at both costs offset (for example, in juvenile justice) and costs saved (for example, by youths remaining in mainstream education) in relation to having received MST or MAU services.

## Trial status

Ongoing. Sites were launched in five phases, once they were well embedded in their local authority and services were familiar with the referral process and eligibility criteria. Recruitment began in February 2010 and recruitment is expected to be complete 18 months after the launch of the last site. Outcome data will be available 18 months post-randomisation of the last participant.

## Abbreviations

ADHD: Attention deficit hyperactivity disorder; CAMHS: Child and adolescent mental health services; CD: Conduct disorder; CI: Confidence interval; CU: Callous-unemotional; CYPRESS: Children and Young People – Resources, Evaluation and Systems Schedule; MAU: Management as usual; MST: Multisystemic therapy; NICE: National Institute for Health and Clinical Excellence; RA: Research assessor; RCT: Randomised controlled trial; SMD: Standardised mean difference; TAM-R: MST Therapist Adherence Measure-Revised; TAU: Treatment as usual; YOT: Youth offending team.

## Competing interests

The authors declare that they have no competing interests.

## Authors’ contributions

PFo is Chief Investigator on the START Trial and drafted the manuscript. SBu is Project Coordinator on the trial. PFo, SBu, PFu, SS, IE, and SP will oversee data collection from five pilot sites from the London hub. IG will oversee one pilot site from the Cambridge hub. DC and AK will oversee four pilot sites from the Leeds hub. SBy will carry out the health economic analysis. JW designed and will perform the statistical analysis. RH is Trial Coordinator. All authors participated in the review and revision of the manuscript and approved the final manuscript.
